# Two-Phase Distributed Genetic-Based Algorithm for Time-Aware Shaper Scheduling in Industrial Sensor Networks

**DOI:** 10.3390/s26020377

**Published:** 2026-01-06

**Authors:** Ray-I Chang, Ting-Wei Hsu, Yen-Ting Chen

**Affiliations:** Department of Engineering Science and Ocean Engineering, National Taiwan University, Taipei 10617, Taiwan; f11525125@ntu.edu.tw

**Keywords:** Time-Sensitive Networking (TSN), IEEE 802.1Qbv, Time-Aware Shaper (TAS), genetic algorithm (GA), Industry 4.0, edge computing

## Abstract

Time-Sensitive Networking (TSN), particularly the Time-Aware Shaper (TAS) specified by IEEE 802.1Qbv, is critical for real-time communication in Industrial Sensor Networks (ISNs). However, many TAS scheduling approaches rely on centralized computation and can face scalability bottlenecks in large networks. In addition, global-only schedulers often generate fragmented Gate Control Lists (GCLs) that exceed per-port entry limits on resource-constrained switches, reducing deployability. This paper proposes a two-phase distributed genetic-based algorithm, 2PDGA, for TAS scheduling. Phase I runs a network-level genetic algorithm (GA) to select routing paths and release offsets and construct a conflict-free baseline schedule. Phase II performs per-switch local refinement to merge windows and enforce device-specific GCL caps with lightweight coordination. We evaluate 2PDGA on 1512 configurations (three topologies, 8–20 switches, and guard bands $\delta_{gb}\in \{0,\;100,\;200\}\;\mathrm{ns}$). At $\delta_{gb}=0$ ns, 2PDGA achieves 92.9% and 99.8% CAP@8/CAP@16, respectively, compliance while maintaining a median latency of 42.1 μs. Phase II reduces the average max-per-port GCL entries by 7.7%. These results indicate improved hardware deployability under strict GCL caps, supporting practical deployment in real-world Industry 4.0 applications.

## 1. Introduction

Time-Sensitive Networking (TSN) has emerged as a critical enabler for deterministic communication within Industrial Sensor Networks (ISNs), bridging traditional Information Technology (IT) and real-time Operational Technology (OT) environments [1]. By incorporating precise time synchronization and scheduled transmission mechanisms, TSN guarantees bounded latency and minimal jitter—essential properties for applications in ISNs, including factory automation, robotics, and process control systems.

Among various TSN standards, IEEE 802.1Qbv, which defines the Time-Aware Shaper (TAS), has received significant attention due to its capability to enforce precise time-triggered schedules. TAS achieves deterministic latency by scheduling the opening and closing of gates at network switches according to pre-defined time schedules, thereby controlling traffic flows strictly and predictably [2]. Deterministic end-to-end latency is particularly crucial in distributed sensor and control networks, where even minor timing violations can negatively impact system safety and performance [3].

A core challenge in the practical deployment of TSN using TAS is the generation of Gate Control Lists (GCLs) for network switches. Each GCL specifies the precise timing of traffic gate operations for all time-triggered traffic flows. Formulating a globally feasible schedule that meets strict timing constraints for all flows is inherently complex, representing a combinatorial optimization problem proven to be NP-hard [4]. Consequently, computational complexity escalates rapidly with network size and the number of scheduled flows, making exhaustive searches impractical for large-scale networks.

Currently, most TSN deployments rely on a Centralized Network Configuration (CNC) entity for schedule computation. The CNC aggregates flow requirements and calculates a synchronized GCL for each network node. Although centralized approaches can achieve optimal schedules for smaller networks, they exhibit substantial scalability limitations. Specifically, the computational burden of a centralized scheduler significantly increases as the network expands, creating a bottleneck that limits the feasible network size [5]. Moreover, the centralized architecture concentrates the computational load on a single node, which may become inefficient for the large-scale topology management.

The limitations of centralized scheduling methods become increasingly prominent in large-scale ISNs. While centralized solvers can optimize globally, they often struggle to address local device constraints efficiently. ISNs often comprise heterogeneous devices, each with distinct hardware limitations—for example, some economical TSN-capable devices support limited GCL entries due to memory and hardware constraints [6]. Centralized methods struggle to accommodate such diversity, potentially resulting in conservative schedules that under-utilize network resources. Additionally, the geographical dispersion of sensor nodes further exacerbates the inefficiency and latency associated with centralized control schemes, motivating the exploration of distributed solutions that share scheduling responsibilities across the network nodes.

**Figure 1:**
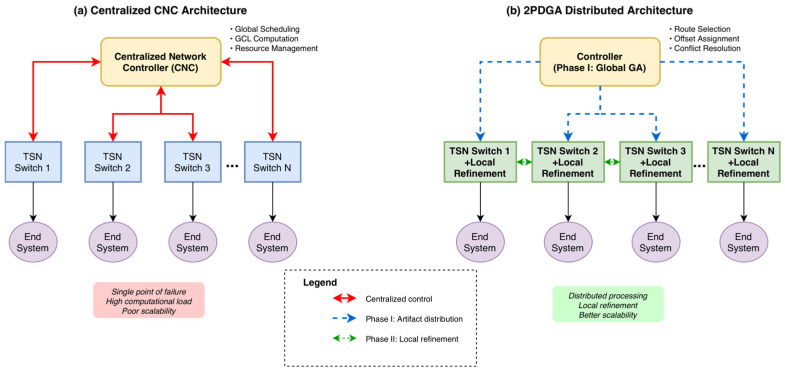
Centralized CNC vs. 2PDGA Distributed Architecture for IEEE 802.1Qbv TSN.

In response to these challenges, this paper introduces 2PDGA, a Two-Phase Distributed Genetic-Based Algorithm specifically designed to address TAS scheduling within IEEE 802.1Qbv networks in a decentralized manner. Figure 1 contrasts a centralized CNC architecture with the proposed two-phase distributed design: (a) the CNC concentrates global scheduling, GCL computation, and resource management, whereas (b) 2PDGA performs Phase I globally (route selection, offset assignment, conflict resolution) and Phase II locally at each switch via a heuristic-based local optimizer to synthesize per-port GCLs and refine schedules, thereby improving computational efficiency and GCL compactness. The proposed 2PDGA approach divides scheduling into two complementary phases: a global genetic algorithm (GA) phase, which provides a coarse-grained optimization of end-to-end latency and preliminary scheduling across the entire network, and a distributed heuristic refinement phase, where individual network nodes independently adjust their local GCLs based on device-specific constraints and fine-tune their schedules. This hierarchical, distributed structure distributes the scheduling workload across network nodes, thereby enhancing scalability and facilitating parallel processing.

To assess practicality under hardware constraints, we evaluate 2PDGA in TSNKit on 1512 configurations spanning three topologies (Chain/Star/Grid), seven network sizes (8–20 switches), and guard bands ($\delta_{gb}\in \{0,\;100,\;200\}\;\mathrm{ns}$). Using CAP@N to represent strict per-port GCL entry limits, 2PDGA achieves 92.9% CAP@8 and 99.8% CAP@16 at $\delta_{gb}=0\;\mathrm{ns}$, and maintains 97.6–98.0% CAP@16 at $\delta_{gb}=100$–200 ns. At $\delta_{gb}=0$ ns, the measured median/P99 end-to-end latency is 42.1/1409.8 μs. We further quantify the impact of Phase II, which reduces the average max-per-port entry count from 6.02 to 5.55 (−7.7%) over all guard-band settings (*n* = 1512). For $\delta_{gb}=0$ ns, inter-switch coordination is required in 32/504 cases (6.3%), with 545 bytes of control traffic per case on average.

The main contributions of this paper are summarized as follows. (1) We present 2PDGA, a two-phase distributed TAS scheduling framework for IEEE 802.1Qbv, which combines a network-level GA (Phase I) with per-switch local refinement (Phase II) to effectively decouple global feasibility search from local hardware adaptation. (2) We design a cap-aware per-switch refinement procedure that specifically targets GCL fragmentation and enforces per-port entry caps; in our evaluation, Phase II is shown to reduce average max-per-port entries from 6.02 to 5.55 (−7.7%) with an average communication overhead of 545 bytes. (3) We conduct a comprehensive evaluation across 1512 configurations and varying guard bands ($\delta_{gb}\in \{0,\;100,\;200\}\;\mathrm{ns}$), demonstrating that 2PDGA achieves 92.9% CAP@8 and 99.8% CAP@16 at $\delta_{gb}=0\;ns$, maintains 97.6–98.0% CAP@16 at $\delta_{gb}=100$–200 ns, and reports detailed end-to-end latency statistics (e.g., 42.1/1409.8 μs median/P99 at $\delta_{gb}=0$ ns).

The remainder of this paper is organized as follows: Section 2 reviews related work on TAS/TSN scheduling, GA-based methods, and distributed scheduling approaches. Section 3 introduces the system model and formalizes the TAS scheduling problem and constraints. Section 4 describes 2PDGA, including Phase I global GA design and Phase II per-switch refinement. Section 5 details the experimental setup, baselines, and evaluation metrics. Section 6 presents experimental results, including CAP@N compliance, guard-band sensitivity, topology effects, scalability, and communication overhead. Section 7 concludes the paper and outlines future work.

## 2. Related Works

### 2.1. Overview of TSN Scheduling Algorithms

Precise scheduling algorithms are essential for deterministic communication in TSN, particularly when employing the IEEE 802.1Qbv TAS. The scheduling techniques for TAS generally fall into two categories: exact optimization methods and heuristic approaches.

Exact methods include Integer Linear Programming (ILP), Constraint Programming (CP), and Satisfiability Modulo Theory (SMT). These approaches theoretically guarantee optimal solutions under defined constraints [4]. However, TAS scheduling has been shown to be NP-hard, implying that the complexity exponentially grows with network scale and flow numbers [4,5]. Consequently, exact methods become computationally infeasible for large networks. For instance, Falk et al. [7] demonstrated the practical limitations of ILP-based scheduling methods, highlighting significant scalability issues when network size increases. Similarly, CP-based methods improve efficiency by using higher-level constraints (e.g., “all-different” constraints), yet still face scalability challenges [8].

To overcome scalability issues, heuristic methods have been widely adopted. Such algorithms, while not guaranteeing optimality, rapidly provide high-quality feasible schedules suitable for practical use. Common approaches include List Scheduling (LS) and Conflict Graph (CG) algorithms. LS employs a greedy mechanism, sequentially allocating feasible transmission slots along the routing path for each flow [9]. CG abstracts transmissions into graph nodes, where conflicts (e.g., simultaneous transmissions on the same link) are represented as edges. Feasible schedules correspond to independent sets in this graph [10]. In practice, conflict-graph-based solvers combine heuristic search with ILP calls, significantly reducing computational complexity compared to solving full ILP formulations directly.

### 2.2. TSNKit Tool and Benchmark Algorithms

To facilitate evaluation and comparison, the community has developed benchmark tools such as TSNKit, an open-source Python toolkit designed for configuring and scheduling in TSN environments [11]. TSNKit incorporates various well-established algorithms, providing researchers with standardized baselines. In this study, all TSNKit-based baselines were executed using TSNKit v0.2.0 with Python 3.10.18.

TSNKit supports exact methods, including ILP-based joint routing and scheduling (JRS), which simultaneously computes optimal paths and transmission schedules under strict end-to-end latency constraints. Such ILP formulations, while optimal, are practical only for smaller networks due to their computational complexity [7]. TSNKit also implements a no-wait scheduling baseline (SMT-NW), which models TAS synthesis under a no-wait constraint. Following Dürr and Nayak [12], this baseline can be solved via an ILP formulation and is commonly enhanced with a Tabu-search heuristic to improve scalability.

Additionally, TSNKit provides both LS and CG. LS assigns transmission slots incrementally along each route to minimize computational overhead [9]. In contrast, CG constructs a configuration–conflict graph in which vertices represent candidate transmission configurations and edges represent mutual exclusions; feasible schedules correspond to independent vertex sets in this graph, which TSNKit computes following the conflict-graph method of Falk et al. [10]. These diverse algorithms serve as essential benchmarks to validate the proposed 2PDGA.

### 2.3. Genetic Algorithms in Scheduling Applications

Genetic algorithms (GAs), a subset of evolutionary computing, are widely recognized for their robustness in solving complex optimization and scheduling problems. In the context of TSN, GA-based methods have demonstrated considerable promise in scheduling efficiency and constraint satisfaction. Several works have employed GA for JRS optimization. Pahlevan and Obermaisser [13] proposed a GA-based scheduler for TSN, encoding routing paths and transmission slots into chromosomes and evaluating solutions through customized fitness functions. This approach demonstrated enhanced schedulability compared to traditional heuristics. Similarly, Kim et al. [14] applied GA to automotive Ethernet TSN scheduling, optimizing for multiple performance metrics such as end-to-end latency, jitter, and bandwidth usage. Their results indicated that GA substantially reduced latency and timing deviations while satisfying critical real-time constraints.

Researchers have further enhanced GAs by combining them with other optimization strategies, notably Particle Swarm Optimization (PSO). Zheng et al. [15] developed a hybrid GA-PSO algorithm that periodically integrates PSO operators to enhance population diversity and convergence rates. Their results showed that hybrid approaches could significantly outperform traditional GAs, reducing total transmission delays by approximately 8%. Despite these advances, existing GA-based approaches primarily optimize for latency or schedulability without explicitly considering hardware deployment constraints. This gap between algorithmic output and hardware capability motivates the design of 2PDGA, which indirectly promotes GCL compaction through offset clustering and path selection in Phase I, and explicitly enforces hardware caps in Phase II.

### 2.4. Distributed Scheduling Approaches

Distributed scheduling has proven effective in various network domains for enhancing scalability, fault tolerance, and responsiveness. In ISNs, distributed slot allocation protocols enable local coordination among nodes, minimizing the computational burden and the risk associated with single-point failures inherent in centralized systems [16]. Similarly, vehicular networks and Multi-access Edge Computing (MEC) scenarios commonly adopt distributed or hierarchical control frameworks, where multiple local controllers coordinate autonomously to manage network resources efficiently [17].

In contrast, standard TSN scheduling currently relies predominantly on CNC entities that compute and disseminate global schedules. This centralized approach introduces latency during network reconfigurations and represents a significant vulnerability due to potential single-point failures [5]. Despite the recognized need for distributed methods, few distributed scheduling solutions for TAS currently exist.

Recent exploratory research has introduced distributed scheduling methods, including reinforcement learning (RL)-based local schedulers embedded in network switches. For example, Zhong et al. [18] proposed a RL scheduler for TSN, allowing switches to autonomously adapt scheduling decisions based on local state information, demonstrating the viability of decentralized scheduling concepts. However, such solutions remain preliminary, and comprehensive distributed algorithms for IEEE 802.1Qbv TAS scheduling are still lacking. 2PDGA directly addresses this gap by integrating the global optimization capabilities of GA with localized, distributed refinements, significantly enhancing scalability and hardware efficiency in large-scale TSN deployments.

### 2.5. Comparison with GA-Based TSN Scheduling Algorithms

To deepen the related work discussion, Table 1 compares our proposed 2PDGA against representative genetic-algorithm-based TSN scheduling approaches in the literature. We focus on key dimensions including the scheduling architecture (centralized vs. distributed), primary optimization objectives, consideration of hardware constraints (e.g., GCL entry limits), and the intended application scenarios. This comparison highlights how 2PDGA’s unique design (“global coarse optimization + local hard constraint adaptation”) differs from prior GA-based solutions and clarifies its novel contributions.

As summarized in Table 1, existing GA-based schedulers [13,14,15] predominantly employ a centralized architecture. While these methods successfully optimize for timing metrics and search convergence, they assume idealized hardware capabilities and neglect practical device constraints such as GCL entry limits. Consequently, schedules generated by these global-only approaches may be theoretically feasible but fail deployment on commercial switches with limited memory.

In contrast, 2PDGA bridges this gap through a novel “global coarse optimization + local hard constraint adaptation” strategy. By decoupling global routing decisions (Phase I) from local hardware compliance enforcement (Phase II), 2PDGA explicitly addresses GCL entry caps and reduces schedule fragmentation at the device level. This distributed refinement ensures that the resulting schedules are not only optimized for latency but are also practically deployable on resource-constrained hardware, addressing a critical limitation in prior centralized GA methods.

## 3. System Model and Problem Statement

This section formalizes the scheduling problem solved by our 2PDGA. We first introduce the network model used by our simulator, then describe TAS, state the optimization objective and feasibility constraints, and finally recall the computational hardness of timetable synthesis. Throughout this section, we assume that all devices in the TSN are synchronized to a global clock using IEEE 802.1AS. To facilitate the description of the network model and the proposed scheduling algorithm, we summarize the key mathematical notations and parameters used in this paper in Table 2.

### 3.1. Network Model

A TSN installation is represented by a directed graph $G=\left(V,E\right)$ whose vertices V comprise both end systems and switches. Each directed edge $e=\left(u,v\right)\in E$ corresponds to one direction of a full-duplex Ethernet link between nodes u and v. Every edge is annotated with a constant line-rate, a fixed propagation delay and a per-frame processing delay incurred at the transmitting switch. Because all devices share the same global clock and operate with sub-microsecond synchronization [1], the entire network follows a common cycle length $T_{cycle}$ without drift.

Traffic to be scheduled consists exclusively of time-triggered (TT) flows. A flow $f_{i}$ is defined by the tuple $\left(s_{i},d_{i},L_{i},T_{i},D_{i}\right)$, where $s_{i}$ and $d_{i}$ denote the source and destination vertices, $L_{i}$ is the payload length in bytes, $T_{i}$ is the release period and $D_{i}$ is the end-to-end deadline. The hop sequence $\pi_{i}=\{e_{1},\ldots ,e_{k}\}$ that carries the flow from $s_{i}$ to $d_{i}$ is assumed loop-free and is drawn from a pre-computed set of candidate routes between $s_{i}$ and $d_{i}$. These candidate paths are generated by enumerating loop-free routes and filtering those that satisfy the flow’s end-to-end deadline constraint $D_{i}$.

**Figure 2:**
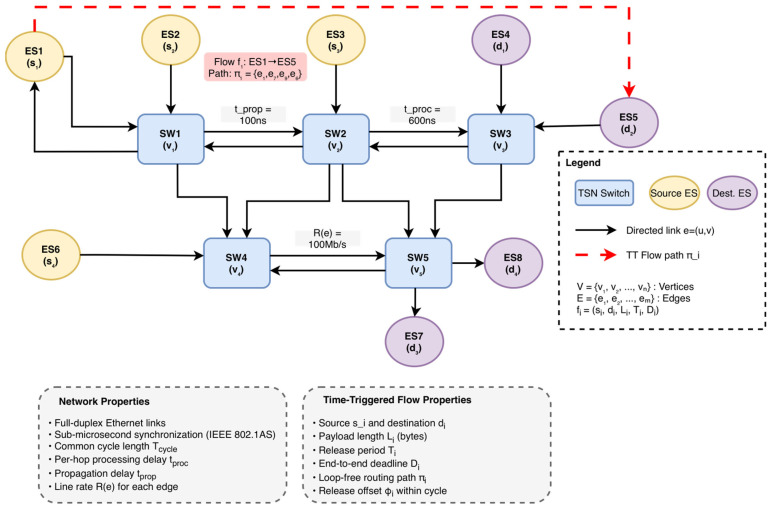
TSN-Network-Model (values shown are for illustration).

Figure 2 illustrates the graph structure and annotations $\left(R\left(e\right),t_{prop},t_{proc}\right)$ and highlights a representative TT path $\pi_{1}$. Directed TSN topology $G=\left(V,E\right)$ with switches and end systems. Directed edges denote link directions; edges are annotated with line rate $R\left(e\right)$, propagation delay $t_{prop}$, and per-frame processing delay $t_{proc}$. The red dashed path shows a representative TT flow along $\pi_{1}$.

### 3.2. IEEE 802.1Qbv Gate-Control Lists

Deterministic forwarding in TSN relies on TAS introduced in IEEE 802.1Qbv. Every egress port of a TSN bridge holds a GCL that repeatedly opens or closes its priority queues at precisely defined instants. Each entry in the GCL specifies a gate state (open or closed) for each queue and the time interval for which that state is active [2]. During an “open” interval packets from the associated queue may be transmitted according to the queue’s priority, whereas during a “closed” interval they are held in the queue [2]. Gate operations are driven by a global clock and repeated every cycle of length $T_{cycle}$; typical cycle times range from a few tens of microseconds to several milliseconds [3].

**Figure 3:**
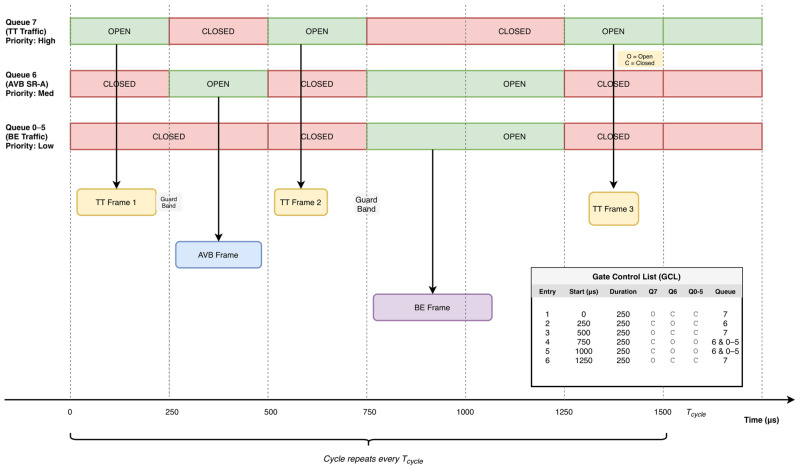
IEEE 802.1Qbv TAS GCL Operation.

Figure 3 visualizes one $T_{cycle}$ and the corresponding six GCL entries of equal duration. Queue 7 (TT) opens in the first, third, and sixth entries; Queue 6 (Audio-Video Bridging (AVB) Stream Reservation Class A (SR-A)) opens in the second entry and in the fourth and fifth entries. The colored arrows indicate that frames are transmitted exclusively within open windows; a small guard band is appended to TT windows to absorb clock error and switchover time. Dashed vertical markers align with GCL entry boundaries, and the table at the right lists the gate states per entry.

A list entry can be written as $\left(t^{start},t^{end},q\right)$, meaning that the queue q alone may transmit in the half-open interval $\left[t^{start},t^{end}\right)$. For a TT frame of size $L_{i}$ to fit entirely into a window, the window length must exceed the serialization time plus a guard band:

(1) $$t^{end}-t^{start}\geq \frac{8L_{i}}{R\left(e\right)}+\delta_{gb}$$

Because the pattern is periodic, each flow leaves its source at a fixed offset $\phi_{i}\in \left[0,T_{i}\right)$. We define the transmission time on edge e as $t_{trans}\left(f_{i},e\right)={8L}_{i}/R\left(e\right)$. The end-to-end latency is calculated as the sum of queuing, transmission, propagation, and processing delays along the path:

(2) $$Latency\left(f_{i}\right)=\sum_{e\in \pi_{i}}\left(t_{queue}\left(f_{i},e\right)+t_{trans}\left(f_{i},e\right)+t_{prop}\left(e\right)+t_{proc}\left(e\right)\right)$$

Two families of constraints must hold simultaneously for a timetable to be feasible. First, each flow must satisfy the deadline constraint:

(3) $$Latency\left(f_{i}\right)\leq D_{i}$$

Second, transmissions must be conflict-free: if two flows share an edge, their windows on that edge may not overlap, and on every intermediate switch the downstream window must open strictly after the upstream transmission and processing finish, thereby preserving causality.

### 3.3. Computational Complexity

Timetable synthesis for TAS is computationally intractable. A classical reduction maps the no-wait job-shop scheduling problem to TAS scheduling: jobs correspond to flows, machines to directed links, and the no-wait constraint mirrors the causality condition. Since the job-shop scheduling problem is a well-known NP-hard optimization problem [4], deciding TAS feasibility is also NP-complete, and minimizing the worst-case latency as posed in (2) is NP-hard. Consequently the search space grows exponentially with the number of flows and hops, and exhaustive enumeration becomes infeasible even for moderate instance sizes. These complexity barriers motivate the meta-heuristic design of 2PDGA.

## 4. Two-Phase Distributed Genetic-Based Algorithm (2PDGA)

This section introduces 2PDGA for synthesizing GCLs under IEEE 802.1Qbv. The algorithm separates the search into a network-wide global phase and a device-local refinement phase. In Phase I, a lightweight genetic search jointly selects a candidate route from a bounded set and a release offset within the common cycle. The outcome is a feasible, interference-free baseline that already respects link mutual exclusion and hop-to-hop causality. In Phase II, each switch independently improves its local windows in parallel to remove fragmentation, reduce the number of GCL entries, and accommodate hardware entry caps while preserving feasibility. The two phases exchange information only through compact artifacts (GCL, ROUTE, OFFSET, QUEUE, and DELAY). Time is discretized into slots of length $t_{slot}$, periods $T_{i}$ are measured in slots, and the cycle length equals the least common multiple (LCM) of all periods. For causality, a conservative per-hop processing delay $t_{proc}^{max}$ is applied uniformly across devices in the global phase. We discretize time into slots of length $t_{slot}=100\;ns$ to convert the continuous-time search space into a finite domain manageable by the GA. The maximum alignment overhead is one slot (≤100 ns), which is <10% of the serialization time of a 128-byte frame at 1 Gb/s.

**Figure 4:**
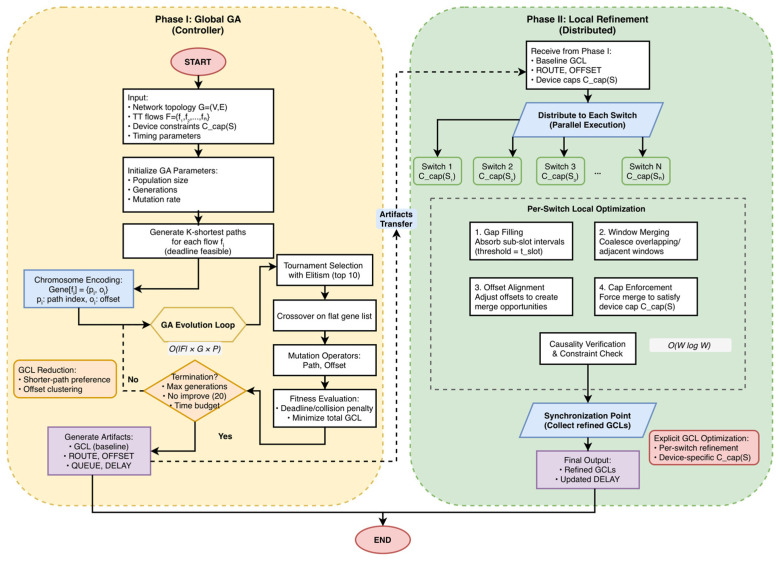
2PDGA flowchart where G denotes the number of GA generations and P denotes the population size.

Figure 4 summarizes the control/data flow, the artifacts exchanged between phases, and annotates the dominant costs near each loop (global $O\left(\left|F\right|\times G\times P\right)$, local $O\left(W\log W\right)$).

Phase I (left) runs a global GA to produce a feasible baseline schedule and emits artifacts {GCL, ROUTE, OFFSET, QUEUE, DELAY}. Phase II (right) performs per-switch local refinement—gap filling, window merging, entry-cap enforcement, and causality verification—in parallel, followed by a synchronization step. Complexity annotations indicate the total cost of the GA loop $O\left(\left|F\right|\times G\times P\right)$ and the dominant per-switch workload $O\left(W\log W\right)$.

### 4.1. Phase I—Global GA

Phase I (Figure 4, left) aims to generate a network-wide baseline timetable that eliminates conflicts on each directed link and minimizes the end-to-end latency over one cycle. The chromosome for a flow $f_{i}$ contains two genes: a path index $p_{i}$ and a release offset $o_{i}$.

The path index refers to a small, pre-computed candidate set of loop-free routes built by K-shortest enumeration under a hop bound. The release offset $o_{i}$ is defined in $\left[0,T_{i}\right)$ and, in implementation, is handled modulo the cycle length for efficient vectorized reduction. Given a chromosome, the evaluator constructs, for each link, a Boolean timeline representing occupancy within the cycle. Each hop advances by the sum of serialization time and the conservative processing delay, yet only the serialization portion occupies the link timeline; the trailing processing segment enforces the no-wait causality between consecutive hops. If any occupied slice overlaps with existing occupancy on the same link, the individual is deemed infeasible and receives a large penalty. The fitness function penalizes deadline violations and minimizes average end-to-end latency. Beyond explicit optimization, Phase I incorporates two design choices that implicitly reduce GCL fragmentation: (1) path selection strongly favors shorter routes, reducing the number of hops per flow; and (2) release offsets are constrained to a small fraction of the period, causing transmissions to cluster temporally rather than scatter across the cycle. This clustering naturally produces adjacent windows on shared links, enabling consolidation without requiring an explicit entry-count term in the fitness function—which would significantly increase per-candidate evaluation cost.

The genetic configuration follows a standard yet tuned setting for TSN instances. In our experiments, we set the population size to 120 with 180 generations per restart. The algorithm performs up to 10 independent restarts within an 8 s time budget. Selection uses tournament sampling (k = 3) with elitism preserving the top 10 individuals. Crossover with per-gene exchange probability of 0.5 promotes structural mixing. Mutation operators include path mutation (20% probability) and offset perturbation (35% probability) with modular reduction to respect the bounds of both the path index and the release offset. The crossover rate is set to 0.8 and mutation rate to 0.2. The evaluator is implemented with vectorized operations to keep the per-generation cost low even for hundreds of flows. An early-stopping rule terminates evolution when the best fitness has stalled for 20 consecutive generations, which preserves quality while keeping runtime modest on larger instances.

**Figure 5:**
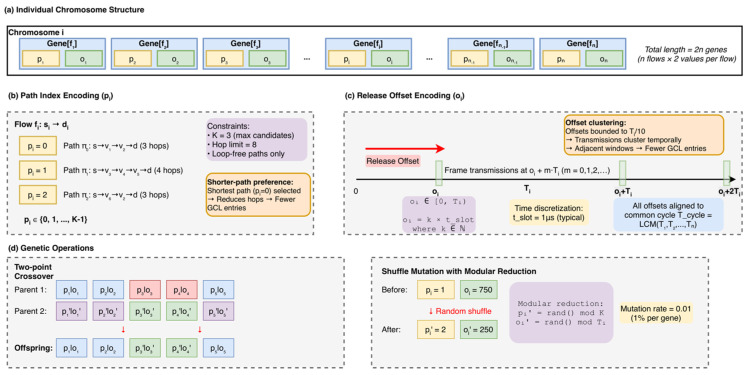
Chromosome Encoding Structure for 2PDGA Phase I.

Figure 5 illustrates the chromosome encoding structure and genetic operators designed for the Phase I global search. (a) An individual chromosome is organized as an ordered sequence of gene pairs $\left(p_{i},o_{i}\right)$ for n flows, resulting in a total length of 2n genes. (b) The path-index encoding ($p_{i}$) selects a routing path from a set of up to K pre-computed loop-free candidates subject to a hop bound. (c) The release-offset encoding ($o_{i}$) determines the transmission start time as a discrete multiple of the time slot $t_{slot}$ within the range $\left[0,T_{i}\right)$, aligned to the common network cycle $T_{cycle}=\mathrm{LCM}\left(T_{1},\ldots ,T_{n}\right)$. (d) Genetic operators include crossover, which uses even-index cut points to preserve the integrity of $\left(p_{i},o_{i}\right)$ pairs, and shuffle mutation with modular reduction applied at a low probability (0.2) to maintain constraint validity.

Upon completion, Phase I emits five artifacts that fully describe the baseline schedule. The GCL file lists the queue state, start time, end time, and cycle length for each directed link in nanoseconds; by construction, the marked duration equals the serialization time. The ROUTE file records the set of hops used by each flow, while the OFFSET file stores the per-flow hold time from which the original release offset can be recovered. The QUEUE file records queue assignments for each hop; in our experiments, a single time-triggered queue per port is used. Finally, the DELAY file reports the end-to-end latency achieved by the baseline timetable. Using a uniform processing delay $t_{proc}^{max}$ strikes a safe balance: it may slightly over-approximate some hops on heterogeneous hardware, but it greatly simplifies causality checks in the global search.

### 4.2. Phase II—Local Refinement

While Phase I produces schedules with reduced fragmentation through implicit design choices, the resulting GCLs may still exceed hardware entry limits on resource-constrained switches. Phase II explicitly enforces hardware compliance by refining the baseline timetable using only local information, running concurrently across all switches. It employs an iterative local search procedure with domain-specific heuristics, operating on each switch independently. From the global GCL, the algorithm constructs, for each switch, a dictionary that groups windows by incident directed link; windows are converted to slot indices and sorted.

A dedicated thread invokes a local optimizer on this per-switch structure to reduce fragmentation and enforce the per-port hardware entry cap. In our evaluation, each egress port uses a single TT queue; therefore, Phase II compaction operates on TT gate-open intervals only and does not require changing queue-state vectors. The optimizer applies monotone transformations on each port timeline. The overall Phase II per-switch refinement workflow is summarized in Algorithm 1.

First, a gap-filling pass (GAP_FILL) absorbs sub-slot (or very small) idle intervals by extending/aligning neighboring TT intervals within a bounded tolerance (t_slot), effectively turning near-adjacent fragments into overlapping or contiguous intervals. Second, a merging pass (GREEDY_MERGE) coalesces overlapping/contiguous TT intervals on the same port into a single longer interval, thereby reducing the number of GCL entries. Any timing changes introduced by local alignment/merging are validated by the end-to-end delay replay and causality verification described later in this section. Third, when the number of entries on a port exceeds the device cap, the optimizer greedily coalesces the nearest compatible windows or the smallest-gap pairs until the cap is met; if a merge would invert causality, the optimizer defers that merge and prefers alternatives with minimal slack loss.

Because Phase II may shift or merge windows, end-to-end delays are recomputed over the refined lists. The ROUTE artifact is used to reconstruct the unique ordered path of each flow by inferring the successor and predecessor relationships within the set of unordered hops. The OFFSET artifact recovers the original release offset from the stored hold time and the known period. Forward propagation then proceeds hop by hop: for each directed link, the algorithm selects the first window whose start is not earlier than the current time modulo the cycle, wrapping to the next cycle only when necessary, and advances the clock to the end of that window plus the conservative processing gap. The resulting end-to-end latency equals the final time minus the recovered offset and the trailing processing delay. This recomputation is purely local to the GCLs and does not require re-invoking the global genetic search. To ensure end-to-end causality is maintained across the network, communication among devices is limited to compact summaries for the flows whose windows changed, so the message volume grows only with the number of affected flows and their hop counts. With sorted lists, the dominant work per switch is the initial ordering of windows, which runs in $O\left(W\log W\right)$ where W is the number of local windows; linear passes suffice for the three transformations. Since each switch optimizes independently, wall-time is governed by the largest local workload rather than the sum over the entire network.

**Algorithm 1:** Phase II—Per-Switch Local Refinement**Input**: Baseline GCL from Phase I, device-specific caps C_cap(S) **Output**: Refined GCLs satisfying hardware entry limits per switch   1: function PHASE_II_REFINE(GCL, {C_cap(S)}) 2:   // Distribute to switches and refine in parallel  3:   parallel for each switch S do  4:    windows ← PARTITION_BY_PORT(GCL, S)  5:    for iter = 1 to I do              // I = 8  6:     for each port P in S.egress_ports do  7:      if COUNT_ENTRIES(windows[P]) ≤ C_cap(S) then continue  8:  9:         // Step 1: Gap filling-absorb sub-slot intervals 10:      windows[P] ← GAP_FILL(windows[P], t_slot) 11: 12:      // Step 2: Greedy merge-coalesce compatible windows 13:      windows[P] ← GREEDY_MERGE(windows[P]) 14: 15:      // Step 3: Offset alignment-adjust timing for merging 16:      if COUNT_ENTRIES(windows[P]) > C_cap(S) then 17:       windows[P] ← ALIGN_OFFSETS(windows[P], C_cap(S)) 18: 19:      // Step 4: Cap enforcement-force merge if needed 20:      if COUNT_ENTRIES(windows[P]) > C_cap(S) then 21:         windows[P] ← FORCE_MERGE_TO_CAP(windows[P], C_cap(S)) 22: 23:    GCL_refined[S] ← RECONSTRUCT(windows) 24: 25:   // Synchronization and delay recomputation 26:   return MERGE_ALL(GCL_refined), RECOMPUTE_DELAYS() 27: function GREEDY_MERGE(windows) 28:     // Sweep-line merge of overlapping/contiguous TT intervals after GAP_FILL/ALIGN_OFFSETS 29:   windows ← SORT_BY_START(windows) 30:   merged ← [] 31:   for each (st, en) in windows do 32:    if merged is not empty and st ≤ merged[-1].end then 33:     merged[-1].end ← max(merged[-1].end, en) 34:    else 35:     merged.append((st, en)) 36:   return merged

### 4.3. Rescue Search (Fallback Repair)

Rescue Search is a time-bounded fallback invoked only when the max-per-port GCL count still exceeds the hardware cap after Phase II refinement. It performs a small, conservative local repair (candidate offset alignment and limited path alternatives) and accepts only conflict-free schedules that meet all deadline constraints. This step is not a core contribution; it is a safety net used sparingly when cap violations remain.

## 5. Experimental Setup

This section details the simulation environment, network topologies, traffic workloads, baseline algorithms, and evaluation metrics used to assess the performance of the proposed 2PDGA. The experimental design is structured to rigorously evaluate the algorithm’s scalability, GCL efficiency, and hardware compliance against both heuristic and solver-based scheduling methods.

### 5.1. Software and Hardware Environment

Experiments were conducted on a Linux workstation running Ubuntu 24.04 LTS, equipped with a 13th Gen Intel^®^ Core™ i7-13700K CPU (24 threads, up to 5.4 GHz) and 64 GB of RAM. All algorithms were implemented and evaluated using TSNKit, an open-source TSN scheduling toolkit. To ensure reproducibility and determinism, fixed random seeds were used across all trials, ensuring that all algorithms were evaluated on identical problem instances.

### 5.2. Network Models and Traffic Workloads

The simulator adopted the standard TSN abstraction of a directed graph $G=\left(V,E\right)$ assuming IEEE 802.1AS global synchronization. All links operated at 1 Gb/s. To reflect the realistic forwarding latency of store-and-forward switches used in our model, we applied a per-frame processing delay $t_{proc}=2000$ ns and assumed a negligible propagation delay ($t_{prop}\approx 0$ ns) typical of short-range industrial cabling.

To evaluate robustness against clock synchronization errors, experiments were conducted with three guard band values: $\delta_{gb}\in \{0,\;100,\;200\}\;\mathrm{ns}$. The guard band represents additional idle time inserted between consecutive GCL windows to prevent timing violations due to clock drift.

**Figure 6:**
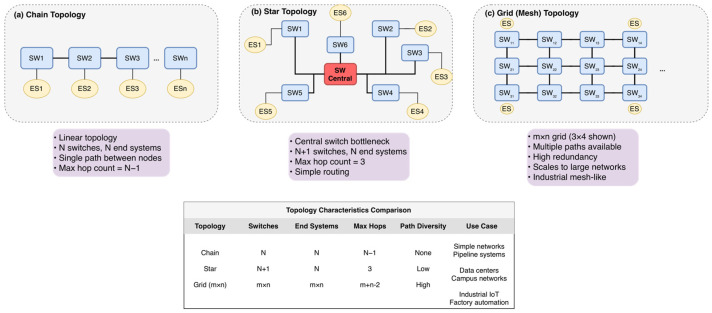
Test network topologies. (**a**) Chain: Linear topology with single-path routing. (**b**) Star: Centralized topology with N+1 switches and low diversity. (**c**) Grid: m×n lattice structure (3×4 shown) offering high redundancy for scalability testing.

To comprehensively evaluate topological adaptability, we investigated three distinct network structures as illustrated in Figure 6. The Chain topology (Figure 6a) represents linear constraints common in automotive daisy-chains, challenging schedulers with accumulated hop delays and a lack of routing diversity. The Star topology (Figure 6b) models centralized industrial cells; while routing is simpler, it creates significant congestion at the central switch bottleneck. Finally, the Grid (Mesh) topology (Figure 6c) represents complex industrial mesh networks (e.g., factory floors). This topology offers high path diversity but presents an exponentially larger search space, serving as the primary stress test for scalability.

To assess scalability, we conducted a parameter sweep of the network size N ranging from 8 to 20 switches. For each size, the number of time-triggered (TT) flows (F) is scaled based on the network size to maintain a consistent traffic density of approximately 0.6 to 0.8 flows per switch. Specifically, the workload follows the relation F=N−4 for N≥12, with baseline flow counts of 5 and 6 for N=8 and N=10, respectively. Traffic attributes are synthesized to mimic realistic industrial workloads. To prevent cycle length (hypercycle) explosion, flow periods are selected from a discrete set (e.g., 0.5, 1, 2, 4 ms) rather than a continuous distribution. To ensure algorithm robustness, we generated diverse traffic configurations by varying flow densities and payload sizes across experiments. The detailed simulation parameters are summarized in Table 3.

In this study, the traffic model consists exclusively of Time-Triggered (TT) flows. While industrial networks typically carry mixed-criticality traffic, we focus on TT traffic based on two key rationales widely adopted in TSN scheduling research. First, ensuring deterministic latency for safety-critical flows is the primary objective of TSN and represents an NP-hard scheduling problem in itself. Focusing on TT flows allows for a rigorous evaluation of the algorithm’s scalability and hardware efficiency without the confounding variables of stochastic traffic, a strategy consistent with established studies such as Geppert et al. [19] and Dürr & Nayak [12]. Second, our model assumes the standard TSN protection mechanisms—Gate Control Lists (GCLs) that close non-TT queues during TT windows, together with guard bands at window boundaries—to protect critical transmission windows [2]. Under this strict isolation configuration, lower-priority AVB/BE frames are prevented from being transmitted (or being in flight) during TT windows; therefore, they do not affect the worst-case timing or feasibility of the TT schedule, allowing the TT synthesis problem to be decoupled and solved independently [4].

### 5.3. Algorithms and Baseline

To validate the effectiveness of 2PDGA, we benchmark it against four representative algorithms spanning heuristic, ILP-based, and solver-assisted approaches. LS [9] is used as a baseline for widely used greedy heuristics; it sequentially assigns the earliest feasible transmission slots, offering low computational cost but no guarantee of optimality. In TSNKit, CG follows Falk et al. and combines a fast heuristic with an ILP-based solver for this independent-set subproblem [10,11], providing a strong (often near-exact) feasibility baseline. We include CG to highlight that constructing a feasible schedule is fundamentally different from producing a hardware-efficient schedule, especially under strict GCL length constraints. ST/BE-Ratio applies traffic-class-aware bandwidth allocation to balance scheduled traffic (ST) and best-effort (BE) flows, representing ratio-based optimization strategies. FlexTAS-Tabu [12,20] is a Tabu-search baseline implementation provided in the FlexTAS artifact [20], following the no-wait packet scheduling (NW-PSP) formulation and Tabu metaheuristic of Dürr and Nayak [12]. For clarity, “FlexTAS-Tabu” in this paper refers to this Tabu baseline (implementation source: FlexTAS), not the FlexTAS scheduling method itself. Note that the released implementation uses a coarse time granularity, which precludes evaluation under non-zero guard band configurations; therefore, we report FlexTAS-Tabu only for $\delta_{gb}=0$.

Regarding the configuration of our proposed 2PDGA, Phase I is initialized with a population size of 120 to ensure sufficient genetic diversity for exploring the solution space, while Phase II executes distributedly to apply local refinements such as gap absorption and window merging. This two-phase configuration ensures that hardware constraints are enforced efficiently without the excessive computational overhead typical of exact methods.

### 5.4. Metrics

We assess performance using the following five metrics. Schedulability Rate (SR) measures the percentage of test cases in which all flows satisfy their end-to-end deadlines.

For GCL Efficiency, consider a network with P egress ports, where $g_{p}$ denotes the number of GCL entries on port p. We report: (i) Total GCL Entries ($\sum_{p=1}^{P}g_{p}$) and Avg GCL, defined as the mean of Total GCL Entries over all test cases; and (ii) the per-test-case maximum entries on any port, $g^{max}=\underset{p}{\max}g_{p}$ together with Avg Max/Port, defined as the mean of $g^{max}$ over all test cases (reported as “Max/Port” in Section 6 for brevity).

Hardware Compliance Rate (CAP@N) evaluates the percentage of valid schedules that satisfy a strict per-port GCL cap N, i.e., $\underset{p}{\max}g_{p}\leq N$. Prior work indicates that practical per-port GCL capacities can range from 8 to 1024 entries depending on device class and implementation constraints [6]. To match the hardware-cap analysis in Section 6, we report CAP@8, CAP@16, and CAP@32. These thresholds reflect representative commercial limits: entry-level platforms such as the TI AM64x ICSSG provide 16 schedule entries [21], mid-range TSN switch solutions such as the Analog Devices ADIN6310 evaluation stack support 32-entry GCLs [22], and industrial switches such as the Moxa TSN-G5008 are commonly evaluated under 64-entry per-port caps [23]. End-to-End Latency is computed for each TT flow from the resulting schedule/GCLs using the end-to-end delay definition in Section 3. We report Median (P50) and P99 latency (in μs) over the distribution of per-flow end-to-end latencies for each algorithm under each guard-band setting $\delta_{gb}$.

Finally, Phase II Contribution quantifies the incremental improvement brought by the distributed refinement stage. Specifically, we measure the change from Phase I to Phase II in terms of Avg Max/Port (and the corresponding CAP@N feasibility), and we additionally report the coordination overhead of Phase II using communication volume statistics (bytes/messages/frames) in Section 6.7.

### 5.5. Evaluation Protocol

For every algorithm-configuration pair, we recorded the generated GCLs, latency statistics, and runtime. Each configuration was tested across all three guard band values (0, 100, 200 ns), yielding 1512 total test cases (504 base configurations × 3 guard bands). We performed paired statistical tests to validate that the observed differences are significant at the 95% confidence level (p<0.05). This rigorous protocol ensures that reported improvements are statistically robust and not artifacts of random variation. For the 2PDGA-specific analysis, we additionally recorded Phase I/II execution times, GA convergence metrics (including generations and the converged generation), and communication overhead measured in bytes, messages, and frames.

## 6. Experiments and Results

This section reports the empirical evaluation of the proposed 2PDGA against four representative baselines: LS, CG, ST/BE-Ratio, and FlexTAS-Tabu. We present a comprehensive analysis spanning 1512 experimental configurations—incorporating varying guard band settings—to assess GCL efficiency, hardware compliance, robustness, and scalability. All reported results are derived from actual experiments with statistical validation.

### 6.1. Overall Performance and GCL Efficiency

**Figure 7:**
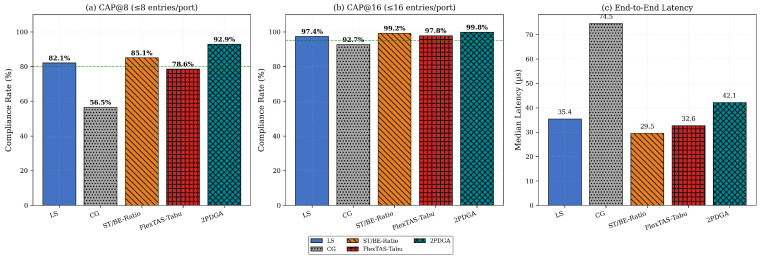
Overall Algorithm Comparison.

We first examine the aggregate performance across all network configurations. Table 4 summarizes the performance for all evaluated algorithms across three guard band values. A total of 1512 experimental configurations (504 per guard band value × 3 guard bands) were evaluated. Figure 7 below provides a visual comparison of key metrics across algorithms. Table 4 reports SR, Avg GCL, Max/Port, CAP@8, CAP@16, and median/P99 latency for each guard band. The figure below (Figure 7) further breaks down these metrics at $\delta_{gb}=0\;ns$ into CAP@8 compliance (Figure 7a), CAP@16 compliance (Figure 7b), and median latency (Figure 7c).

The proposed 2PDGA demonstrates superior GCL compactness and hardware compliance. At $\delta_{gb}=0\;ns$, 2PDGA achieves an average of 45.54 total GCL entries with an avg max-per-port value of 4.998, compared to 63.65/6.401 for LS, 114.52/8.788 for CG, and 49.41/5.998 for FlexTAS-Tabu.

More importantly, 2PDGA achieves the highest CAP@8 compliance rate (92.9%), significantly outperforming all baselines. In comparison, LS achieves 82.1% (a + 10.8% gap), ST/BE-Ratio 85.1% (+7.8%), FlexTAS-Tabu 78.6% (+14.3%), and CG 56.5% (+36.4%).

This high CAP@8 compliance enables deployment on cost-sensitive entry-level TSN hardware where other algorithms fail to achieve adequate compliance rates. Regarding latency, 2PDGA achieves a median latency of 42.1 µs ($\delta_{gb}=0$), which is slightly higher than LS (35.4 µs) and ST/BE-Ratio (29.5 µs). However, the P99 latency of 2PDGA (1409.8 µs) is lower than LS (1694.9 µs), indicating better tail behavior and more predictable worst-case performance. This modest median latency increase is justified by the significant improvement in hardware compliance.

### 6.2. Hardware Compliance and Topology Analysis

**Figure 8:**
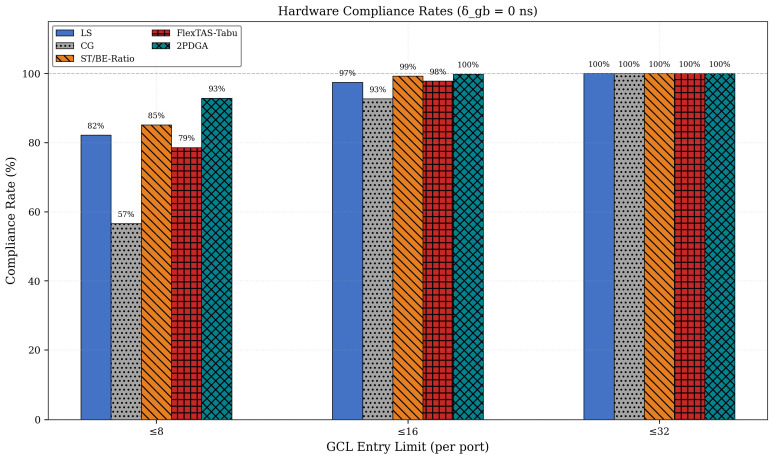
Hardware Compliance Rates.

Under the strict CAP@8 constraint (8 entries per port), 2PDGA achieves a compliance rate of 92.9% at $\delta_{gb}=0$, significantly outperforming all baselines. Figure 8 below illustrates the compliance rates across different GCL entry limits. In particular, Figure 8 below compares CAP@8, CAP@16, and CAP@32 at $\delta_{gb}=0$, highlighting 2PDGA’s consistently highest compliance across thresholds.

As shown in Table 5, 2PDGA maintains the highest CAP@8 compliance across all guard band values. Most algorithms exhibit 10–15% degradation as the guard band increases from 0 to 200 ns, while CG shows minimal sensitivity since its conflict graph structure is unaffected by timing margins. Notably, 2PDGA’s advantage over baselines is preserved even under conservative timing margins, demonstrating its robustness for practical deployment scenarios.

**Figure 9:**
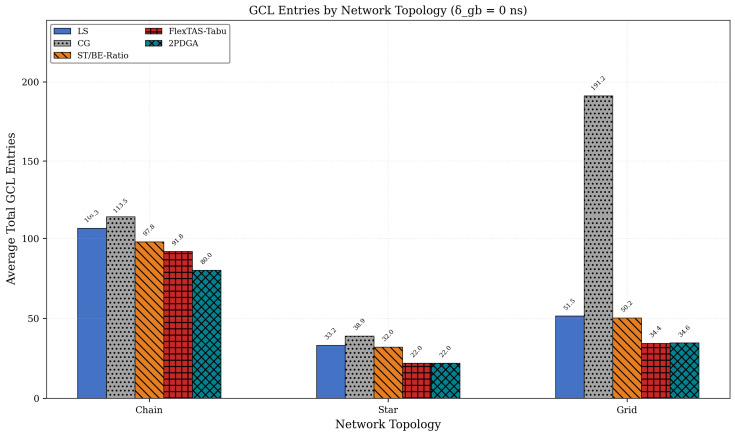
Topology GCL Analysis.

**Figure 10:**
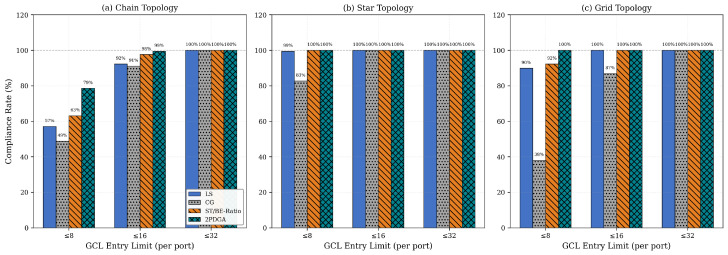
Topology Compliance Rates.

These results demonstrate that 2PDGA enables deployment in hardware-constrained scenarios where other algorithms fail to achieve compliance, providing significant practical value for Industry 4.0 applications with heterogeneous device constraints. Topology-specific behavior is summarized in Table 6 and Figure 9 and Figure 10 below. Table 6 reports the average total GCL entries by topology, while Figure 10 below contrasts CAP@8, CAP@16, and CAP@32 compliance across topologies.

As shown in Figure 9 and Figure 10 below, 2PDGA demonstrates consistent advantages across all topologies. In Chain topology, 2PDGA achieves 78.6% CAP@8 compliance compared to only 48.8% for CG. In Star topology, the simpler network structure allows both 2PDGA and ST/BE-Ratio to achieve 100% CAP@8 compliance. The most dramatic difference appears in Grid topology, where 2PDGA achieves 100% CAP@8 compliance while CG achieves only 38.1%, highlighting 2PDGA’s effectiveness in complex networks with high path diversity. The extreme gap in Grid topology (100% vs. 38.1% CAP@8) demonstrates 2PDGA’s robustness in complex, high-redundancy networks typical of smart factory deployments.

### 6.3. Guard Band Sensitivity Analysis

**Figure 11:**
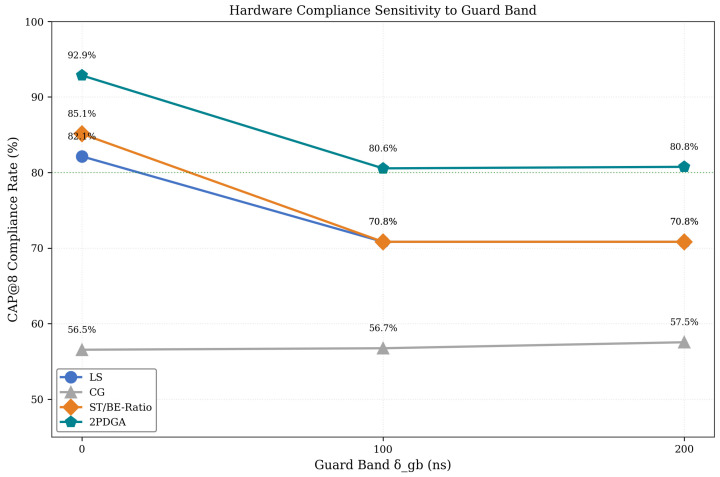
Hardware Compliance Sensitivity to Guard Band.

To evaluate robustness against clock synchronization variations, we tested all algorithms with guard band values of 0, 100, and 200 ns. Figure 11 presents the CAP@8 compliance trend across guard band configurations, with Figure 11 below explicitly comparing $\delta_{gb}=0,\;100,\;200\;ns$ to show how each algorithm’s CAP@8 rate degrades under increasing timing margins.

As shown in Figure 11, all algorithms except CG show degradation with increased guard bands, as larger guard bands reduce the effective window space for scheduling. Despite this trend, 2PDGA maintains over 80% CAP@8 compliance even at $\delta_{gb}=200\;ns$. The distributed baseline ST/BE-Ratio exhibits higher sensitivity (14.3% degradation) compared to 2PDGA (12.1% degradation), demonstrating the robustness of 2PDGA’s optimization approach. Note that FlexTAS-Tabu cannot be evaluated at non-zero guard bands due to its microsecond precision limitation.

These results confirm that 2PDGA’s advantage is not limited to ideal synchronization scenarios but extends to realistic industrial deployments with timing margins.

### 6.4. Ablation: Contribution of Phase II (Hardware Feasibility)

To isolate the impact of Phase II refinement, we conducted an ablation study comparing Phase I output (before window merging and local refinement) against the final optimized results.

Phase II includes two components: window merging, which coalesces adjacent GCL windows serving the TT queue, and per-switch local refinement, which performs gap filling/offset alignment and cap enforcement. Table 7 summarizes how Phase II changes max-per-port GCL entries across all 1512 cases (all guard bands), focusing on hardware feasibility rather than average GCL.

Phase II local refinement is intentionally conservative and activates only when per-port caps are at risk. Across 1512 cases, it reduces max-per-port entries by 0.46 on average, with improvements in 15.3% of cases (3.08 entries when improved). These results indicate that Phase II is intentionally conservative: its average GCL reduction is modest, but its primary contribution is enforcing the per-port max-per-port cap, which directly determines deployability and is reflected by CAP@N improvements in Section 6.2.

### 6.5. Scalability Analysis

**Figure 12:**
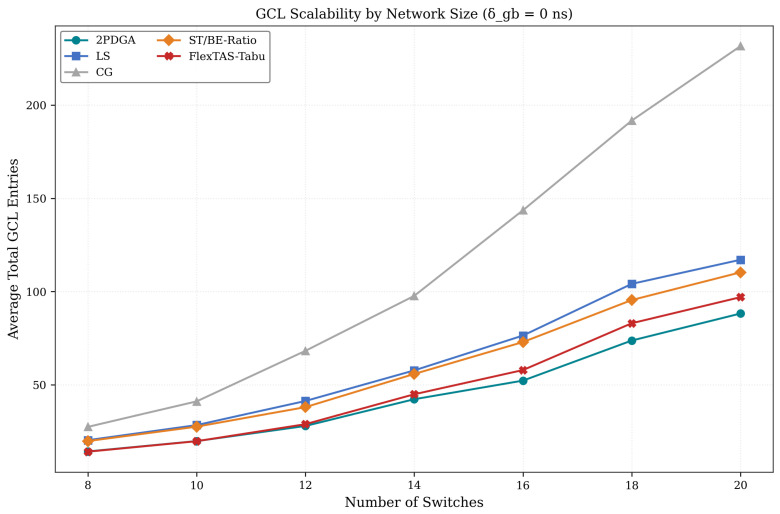
GCL Scalability by Network Size.

We evaluate how algorithm performance scales with network size (8–20 switches). Figure 12 below illustrates the scaling trends for all evaluated algorithms. Table 8 lists the average total GCL entries at each network size, while Figure 12 below visualizes the same trends to highlight relative growth rates across algorithms.

As illustrated in Figure 12, CG exhibits superlinear growth in GCL entries, escalating from 27.5 entries at 8 switches to 231.7 entries at 20 switches (8.4× increase). In contrast, 2PDGA demonstrates moderate growth from 12.8 to 77.8 entries (6.1× increase), while LS and ST/BE-Ratio show approximately linear growth with network size. The relative advantage of 2PDGA over CG remains stable at 53–68% across all network sizes.

The consistent advantage across network sizes confirms 2PDGA’s suitability for large-scale ISN deployments where scalability is critical.

### 6.6. Runtime Analysis

Table 9 presents runtime comparison across all algorithms. We report mean, P95, and max runtime for reference, while noting that direct runtime comparison can be misleading across heterogeneous solver implementations and environments. FlexTAS-Tabu does not support guard bands, so it is excluded from the runtime comparison.

As shown in the phase breakdown Table 10, Phase I accounts for the largest share (45.5%), while Phase II and the fallback Rescue Search (Section 4.3) together contribute only about 10.8%, indicating that local refinement adds limited incremental cost. The remaining 43.6% is dominated by I/O and evaluation steps in the measurement pipeline (schedule serialization, metric aggregation, and latency replay), so the reported wall-clock time should be interpreted as a conservative upper bound; GA evaluation is also naturally parallelizable.

Runtime tables are provided for reference only; differences in solver choice and implementation details can dominate wall-clock time, making direct comparisons not strictly fair. The GA phase is population-based and amenable to parallel evaluation, so the reported wall-clock time is a conservative estimate of achievable runtime under parallel execution. Despite higher runtime, 2PDGA delivers significantly better GCL efficiency.

### 6.7. Communication Overhead Analysis

For practical distributed deployment, Phase II requires inter-switch coordination. We quantify the communication cost to validate deployment feasibility. Table 11 summarizes total bytes, messages, and Ethernet frames across percentiles, providing a compact view of both average and tail communication overhead.

We distinguish two Phase II events: (i) local refinement executed, indicating that Phase II improves the max-per-port GCL entry count within at least one switch, and (ii) inter-switch coordination required, indicating that Phase II triggers cross-switch consensus/verification messages. Table 7 reports event (i) aggregated across all guard-band settings (231/1512 cases, 15.3%), whereas the communication statistics in this section quantify event (ii) for $\delta_{gb}$ = 0 only (32/504 cases, 6.3%).

For $\delta_{gb}$ = 0, the average coordination traffic is 545 bytes per case, which fits within a single Ethernet MTU (1500 bytes). When inter-switch coordination is required, the average volume is 4569 bytes (3–4 frames) with 136.4 messages over 5 refinement rounds. Notably, 93.7% of cases require no inter-switch coordination beyond the initial schedule broadcast, supporting practical distributed deployment with minimal bandwidth consumption.

## 7. Conclusions and Future Work

This paper introduced 2PDGA to address the scalability and efficiency challenges of IEEE 802.1Qbv [24] TAS scheduling. Our extensive empirical evaluation across 1512 configurations (spanning 3 guard band values, 3 topologies, and 7 network sizes) demonstrates that combining genetic algorithm optimization in the global phase with distributed local refinement yields substantial benefits over existing methods. Specifically, 2PDGA achieves 92.9% CAP@8 compliance rate under strict 8-entry hardware constraints ($\delta_{gb}=0$), significantly outperforming LS (82.1%), ST/BE-Ratio (85.1%), FlexTAS-Tabu (78.6%), and CG (56.5%). The algorithm exhibits robust performance across guard band variations (0–200 ns), maintaining over 80% CAP@8 compliance even under conservative timing margins. Phase II refinement improves hardware feasibility by reducing avg max-per-port GCL from 6.02 to 5.55 (−7.7%), while communication overhead remains minimal at less than one Ethernet frame per case on average. The algorithm demonstrates particular strength in complex Grid topologies, achieving 100% CAP@8 compliance where CG achieves only 38.1%. This efficiency is achieved with median latency of 42.1 µs and P99 latency of 1409.8 µs, indicating improved tail behavior relative to LS (1694.9 µs) despite a modest median-latency trade-off. These findings confirm that integrating global genetic search with distributed local refinement is a highly effective strategy for deploying large-scale, hardware-constrained TSNs in Industry 4.0 applications.

## Figures and Tables

**Table 1:** Comparison between 2PDGA and existing GA-based TSN scheduling algorithms.

Algorithm	Architecture	Objectives	Hardware Constraints	Scenario
Pahlevan et al. (2018) [13]	Centralized GA	Minimize makespan & idle gaps; Meet deadlines.	No (Focuses only on theoretical feasibility)	Automotive/Industrial TSN with hard real-time flows.
Kim et al. (2022) [14]	Centralized GA	Optimize latency, jitter, & bandwidth utilization.	No (Assumes ideal hardware; ignores GCL limits)	Automotive Ethernet (In-vehicle networks).
Zheng et al. (2023) [15]	Centralized Hybrid GA + PSO	Enhance search convergence & reduce transmission time.	No (Idealized model; ignores device limitations)	General industrial networks (Mesh/Star topologies).
2PDGA (This Work)	Distributed Two-Phase	Global latency optimization + Local hardware adaptation.	Yes. Explicitly enforces GCL entry caps&reduces fragmentation.	ISNs with resource-constrained switches.

**Table 2:** Summary of Notations.

Symbol	Meaning
$G=\left(V,E\right)$	Directed TSN graph
V	Set of vertices (switches and end systems)
E	Set of directed edges (link directions)
F	Set of time-triggered (TT) flows
$f_{i}$	Flow i: $\left(s_{i},d_{i},L_{i},T_{i},D_{i}\right)$
$s_{i},d_{i}$	Source/destination of $f_{i}$
$L_{i}$	Payload length of $f_{i}$ (bytes)
$T_{i}$	Release period of $f_{i}$
$D_{i}$	End-to-end deadline of $f_{i}$
$\pi_{i}$	Ordered list of edges in the route of $f_{i}$
$e=\left(u,v\right)$	Directed link from node u to v
$t_{proc}$	Per-frame processing delay at a switch
$t_{prop}$	Propagation delay of a link
$T_{cycle}$	Cycle length of the gate-control list
$q_{j}$	Queue opened by the j-th GCL entry
$t_{j}^{start}$	Start time of GCL entry j
$t_{j}^{end}$	End time of GCL entry j
$R\left(e\right)$	Line-rate of edge *e* ($bit\cdot s^{-1}$)
$\delta_{gb}$	Guard band absorbing clock error and switchover
$\phi_{i}$	Release offset of $f_{i}$ in the cycle
$C_{v}$	Maximum number of GCL entries on port v
$Latency\left(f_{i}\right)$	Worst-case end-to-end latency of $f_{i}$
$t_{queue}\left(f_{i},e\right)$	Queuing time of $f_{i}$ on edge e
$t_{trans}\left(f_{i},e\right)$	Transmission time of $f_{i}$ on edge e

**Table 3:** Simulation Parameters and Traffic Configurations.

Parameter	Value/Description
Network Settings	
Link Rate	1 Gb/s
Processing Delay ($t_{proc}$)	2000 ns
Propagation Delay ($t_{prop}$)	0 ns
Network Size (N)	{8, 10, 12, 14, 16, 18, 20} switches
Topologies	Chain, Star, Grid (Mesh)
Traffic Workload	
Flow Count (F)	N−4 (for N≥12); {5, 6} (for N={8, 10})
Flow Period ($T_{i}$)	Discrete: {0.5, 1, 2, 4} ms (scenario-dependent)
Payload Size ($L_{i}$)	256–1024 bytes
Priority	8 queues (0–7)
Algorithm Settings	
2PDGA Population Size	120 (Phase I)
Generations	180
Phase II Iterations	8

**Table 4:** Overall Algorithm Performance Comparison.

Algorithm	$\delta_{gb}$ (ns)	SR (%)	Avg GCL	AvgMax/Port	CAP@8 (%)	CAP@16 (%)	Median (μs)	P99 (μs)
LS	0	100	63.65	6.401	82.1	97.4	35.4	1694.9
CG	0	100	114.52	8.788	56.5	92.7	74.5	1603.1
ST/BE-Ratio	0	100	60.01	5.875	85.1	99.2	29.5	1683.7
FlexTAS-Tabu	0	100	49.41	5.998	78.6	97.8	32.6	1241.7
2PDGA	0	100	45.54	4.998	92.9	99.8	42.1	1409.8
LS	100	100	69.2	7.393	70.8	96.8	26.8	1084.7
CG	100	100	113.54	8.752	56.7	91.5	77.9	1699.5
ST/BE-Ratio	100	100	69.2	7.393	70.8	96.8	44	863.9
2PDGA	100	100	50.1	5.871	80.6	97.6	28.3	984.1
LS	200	100	69.2	7.393	70.8	96.8	28.9	1085.2
CG	200	100	111.47	8.589	57.5	94	67.2	1685.1
ST/BE-Ratio	200	100	69.2	7.393	70.8	96.8	61.7	877.4
2PDGA	200	100	50.03	5.788	80.8	98	28.1	944.1

**Table 5:** CAP@8 Compliance Across Guard Band Values.

Algorithm	CAP@8 ($\delta_{gb}$ = 0)	CAP@8 ($\delta_{gb}$ = 100)	CAP@8 ($\delta_{gb}$ = 200)	Degradation
2PDGA	92.90%	80.60%	80.80%	−12.10%
ST/BE-Ratio	85.10%	70.80%	70.80%	−14.30%
LS	82.10%	70.80%	70.80%	−11.30%
FlexTAS-Tabu	78.60%	N/A	N/A	-
CG	56.50%	56.70%	57.50%	+1.0%

**Table 6:** Topology Performance.

Algorithm	AvgGCL(Chain)	AvgGCL(Star)	AvgGCL(Grid)	CAP@8(Chain)	CAP@8(Star)	CAP@8(Grid)
2PDGA	70.5	19.4	30	78.60%	100.00%	100.00%
LS	106.3	33.2	51.5	57.10%	99.40%	89.90%
CG	113.5	38.9	191.2	48.80%	82.70%	38.10%
ST/BE-Ratio	97.8	32	50.2	63.10%	100.00%	92.30%
FlexTAS-Tabu	91.8	22	34.4	53.60%	94.60%	87.50%

**Table 7:** Phase II Feasibility Impact (Max-Per-Port). Table 7 reports event (i) local refinement executed aggregated over all guard-band settings ($\delta_{gb}$∈0, 100, 200 ns), i.e., 1512 cases = 504 base configurations × 3 guard bands.

Metric	Value
Avg Max/Port (Phase I -> Phase II)	6.02 -> 5.55 (−0.46, −7.7%)
Cases with improved max-per-port after Phase II (all guard bands)	15.3% (231/1512)
Avg reduction when improved	3.08 entries

**Table 8:** Avg Total GCL by Network Size ($\delta_{gb}=0$).

Switches	2PDGA	LS	CG	ST/BE-Ratio	2PDGA vs. CG
8	12.8	20.4	27.5	19.9	−53.50%
10	17.5	28.4	41.1	27.6	−57.40%
12	24.8	41.3	68.2	38	−63.60%
14	37.2	57.7	97.7	55.8	−61.90%
16	46.1	76.4	143.7	72.9	−67.90%
18	65.1	104.2	191.8	95.5	−66.10%
20	77.8	117.1	231.7	110.3	−66.40%

**Table 9:** Runtime Comparison (all test cases).

Algorithm	Mean (ms)	P95 (ms)	Max (ms)
ST/BE-Ratio	586	2081	8890
CG	794	3002	126,536
LS	1195	7474	46,505
2PDGA	1557	6242	35,325

**Table 10:** 2PDGA Phase Runtime Breakdown.

Phase	Time (ms)	Percentage
Phase I (GA)	709	45.50%
Phase II (Local)	113	7.20%
Rescue Search	57	3.60%
Other overhead (I/O, eval)	678	43.60%

**Table 11:** Phase II Inter-switch Coordination Overhead ($\delta_{gb}$ = 0, *n* = 504).

Metric	Average	P50	P90	P99	Max
Total Bytes	545	240	656	7771	8616
Messages	16.7	10	20	226	226
Ethernet Frames	1.16	1	1	6	6

## Data Availability

The original contributions presented in this study are included in the article. Further inquiries can be directed to the corresponding authors.

## Abbreviations

The following abbreviations are used in this manuscript:

2PDGA	Two-Phase Distributed Genetic-Based Algorithm
AVB	Audio-Video Bridging
BE	Best Effort
CAP@N	Hardware compliance rate under a per-port GCL entry cap of N
CG	Conflict Graph
CNC	Centralized Network Configuration
CP	Constraint Programming
GA	Genetic Algorithm
GCL	Gate Control List
ISNs	Industrial Sensor Networks
ILP	Integer Linear Programming
IT	Information Technology
JRS	Joint Routing and Scheduling
LCM	Least Common Multiple
LS	List Scheduling
MEC	Multi-access Edge Computing
MTU	Maximum Transmission Unit
NP	Nondeterministic Polynomial Time
NW-PSP	No-wait Packet Scheduling Problem
OT	Operational Technology
PSO	Particle Swarm Optimization
RL	Reinforcement Learning
SMT	Satisfiability Modulo Theory
SMT-NW	Satisfiability Modulo Theory No-Wait
SR	Schedulability Rate
SR-A	Stream Reservation Class A
ST	Scheduled Traffic
TAS	Time-Aware Shaper
TSN	Time-Sensitive Networking
TT	Time-Triggered
